# Enhancing Within-Person Estimation of Neurocognition and the Prediction of Externalizing Behaviors in Adolescents

**DOI:** 10.5334/cpsy.112

**Published:** 2024-07-26

**Authors:** Sam Paskewitz, Inti A. Brazil, Ilker Yildirim, Sonia Ruiz, Arielle Baskin-Sommers

**Affiliations:** 1 Department of Psychology, Yale University, New Haven CT, US; 2 Radboud University, Donders Institute for Brain, Cognition and Behaviour, Nijmegen, The Netherlands; 3 Forensic Psychiatric Centre Pompestichting, Nijmegen, The Netherlands

**Keywords:** neurocognition, Bayesian latent profile analysis, externalizing behaviors, adolescents

## Abstract

Decades of research document an association between neurocognitive dysfunction and externalizing behaviors, including rule-breaking, aggression, and impulsivity. However, there has been very little work that examines how multiple neurocognitive functions co-occur within individuals and which combinations of neurocognitive functions are most relevant for externalizing behaviors. Moreover, Latent Profile Analysis (LPA), a widely used method for grouping individuals in person-centered analysis, often struggles to balance the tradeoff between good model fit (splitting participants into many latent profiles) and model interpretability (using only a few, highly distinct latent profiles). To address these problems, we implemented a non-parametric Bayesian form of LPA based on the Dirichlet process mixture model (DPM-LPA) and used it to study the relationship between neurocognitive functioning and externalizing behaviors in adolescents participating in the Adolescent Brain Cognitive Development Study. First, we found that DPM-LPA outperformed conventional LPA, revealing more distinct profiles and classifying participants with higher certainty. Second, latent profiles extracted from DPM-LPA were differentially related to externalizing behaviors: profiles with deficits in working memory, inhibition, and/or language abilities were robustly related to different expressions of externalizing. Together, these findings represent a step towards addressing the challenge of finding novel ways to use neurocognitive data to better describe the individual. By precisely identifying and specifying the variation in neurocognitive and behavioral patterns this work offers an innovative empirical foundation for the development of assessments and interventions that address these costly behaviors.

## Introduction

Externalizing behaviors consist of aggressive, rule-breaking, destructive, and deceitful behaviors and are expressed in approximately a third of school-aged youth (Danielson et al., 2021). For many children, externalizing behaviors begin during preschool or early school years. However, children are most at risk for externalizing behaviors during adolescence, a developmental period characterized by significant biological and psychological changes that coincide with increased social challenges (Beauchaine & McNulty, 2013; Moffitt, 1993). Adolescents displaying externalizing behaviors are at increased risk for academic underachievement, family dysfunction, legal system involvement, substance misuse, emotional distress, suicidality, teen pregnancy, and a host of health problems (Bongers, Koot, van der Ende, & Verhulst, 2008; Timmermans, van Lier, & Koot, 2008; Van der Ende, Verhulst, & Tiemeier, 2016). Consequently, externalizing behaviors are associated with a high individual, family, and societal burden; they constitute a leading reason for referral to mental health services among adolescents and are a main cause of disability worldwide (Shepherd & Farrington, 2003). Given the profound burden of externalizing behaviors, determining the factors that underlie these behaviors is an essential step for advancing prevention and treatment development.

### Neurocognition and Externalizing Behaviors

A substantial body of research indicates that neurocognitive differences underlie the onset and maintenance of externalizing behaviors. Neurocognitive models of externalizing behaviors most commonly highlight the relevance of various components of executive functions, a term that encompasses cognitive processes related to the initiation planning, and regulation of behavior (Honrath et al., 2021). Meta-analyses of cross-sectional (Ogilvie, Stewart, Chan, & Shum, 2011) and longitudinal (Yang et al., 2022) data report medium-sized effects showing deficits in executive functioning, particularly within subcomponents of working memory and inhibition. However, neurocognition is a multicomponent construct and other components, such as learning, memory, and general decision-making, also appear deficient among adolescents showing externalizing behaviors (Fairchild et al., 2019; Van Goozen, Langley, & Hobson, 2022). For example, Thompson and colleagues (2019) performed factor analysis on neurocognitive data from a large sample of 9–10-year-olds and found that low scores on the general cognitive ability and learning/memory functions, not just executive functioning, were associated with more externalizing behaviors.

Nonetheless, many studies in this area only include one or two subcomponents of neurocognition and few studies explore components of neurocognition beyond executive functioning. Further, much of the extant research relies on analytic methods (multiple regression, factor analysis) that struggle to capture complex, within-person, interactions among components of neurocognition, despite evidence that neurocognitive components work in concert within each person (Chaku, Barry, Fowle, & Hoyt, 2022; Ruiz, Brazil, & Baskin-Sommers, 2023). Part of the challenge lies in finding effective analytical and conceptual frameworks to better describe variability in neurocognition, both within and across individuals (Brazil, van Dongen, Maes, Mars, & Baskin-Sommers, 2018).

Latent profile analysis (LPA) is a popular method for studying within-person variation. LPA explains observed variables by grouping participants into latent profiles, i.e. categories of people with similar characteristics. In contrast to factor analysis, LPA does not assume homogenous relationships among variables: it can represent groups of participants who perform neurocognitive tasks in different ways. For example, a recent study by Chaku and colleagues (2022) applied LPA to three neurocognitive variables representing different aspects of executive functioning (flanker task for inhibition, list sorting for working memory, and card sorting for cognitive flexibility). They identified four profiles, representing groups with overall high, medium, and low executive function, respectively, as well as one with specifically impaired inhibition. Adolescents belonging to the low executive functioning profile showed more externalizing behaviors than others.

**Figure 1:**
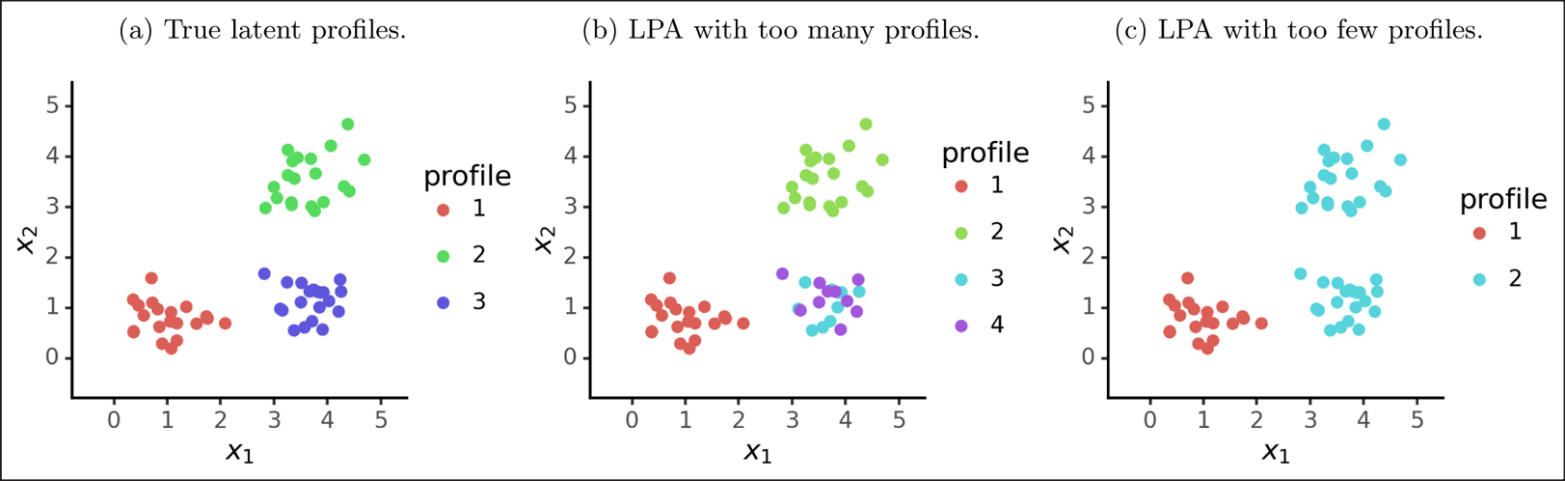
Illustration of LPA with too few or too many profiles.

Despite its advantages for estimating within-person neurocognitive functioning, conventional LPA has a significant limitation: choosing the number of latent profiles involves a tradeoff between model fit and model interpretability (Figure 1 displays a schematic representation). An LPA model with too many latent profiles (Figure 1b) will fit the data well (i.e. have a larger log-likelihood) by producing a fine-grained description of participants. However, this comes at the cost of interpretability: some profiles will be very similar. By comparison, an LPA model with too few latent profiles (Figure 1c) will have distinctive profiles and thus be easy to interpret. However, some participants will not be well described by any of the profiles, resulting in poorer model fit (log-likelihood).

To determine the correct number of latent profiles, researchers fit multiple LPA models, each with a different number of profiles, and compare them using criteria such as model fit, classification uncertainty, a minimum number of people in each profile, statistical tests, and profile interpretability. However, these criteria often disagree, leaving model selection up to the researcher’s judgment (e.g. Chaku et al., 2022; Wendt et al., 2019). The number of profiles selected dramatically influences the interpretation of an LPA model and, by extension, how we understand the relationship between neurocognition and externalizing.

### Using Non-Parameteric Bayesian Inference to Balance the LPA Fit-Interpretability Tradeoff

In the present study, we sought to develop a non-parametric Bayesian LPA model to address limitations of the conventional LPA model and estimate within-person neurocognitive functioning. The Bayesian framework offers a solution by providing flexible probabilistic modeling approaches that rely less on assumptions and conventions and instead, allow us to use data-derived probability distributions to generate inferences (see Van de Schoot et al., 2021, for an introduction).

Bayesian inference uses both the model likelihood and a prior distribution to estimate a model. The prior distribution describes the inherent probability of different model solutions. For LPA, Bayesian inference requires a prior distribution of participants’ latent profile membership. Existing Bayesian LPA methods use a prior that assumes a fixed number of latent profiles (Li, Lord-Bessen, Shiyko, & Loeb, 2018; White & Murphy, 2014). They thus suffer from a similar drawback to conventional LPA: to decide the correct number of latent profiles, one must fit and compare models of different sizes. We avoid this problem by using a distribution called the Dirichlet process (Ferguson, 1973) as the prior on profile membership. The resulting model (a Dirichlet process mixture) is very flexible: it allows for a large number of latent profiles, but places higher prior probability on model fits with fewer profiles. We developed a novel implementation of the Dirichlet mixture for application to latent profile analysis (DPM-LPA). DPM-LPA has the flexibility to infer a large number of latent profiles if needed, but unlike conventional LPA, DPM-LPA favors simpler model fits that produce a small number of distinct, non-redundant profiles (see Method; see also Qiu, Paganin, Ohn, & Lin, 2023, for a computationally different implementation of the same idea).

Here, the first goal was to compare DPM-LPA and conventional LPA. We fit both models to neurocognitive data from 11–12-year-olds included in the Adolescent Brain Cognitive Development Study℠ (ABCD Study®). Every two years participants complete the NIH Toolbox task battery (Luciana et al., 2018) and several other neurocognitive tasks during a magnetic resonance imaging session (Casey et al., 2018). We compared DPM-LPA and conventional LPA with respect to two metrics: profile similarity and the certainty with which participants were classified into profiles (entropy reduction). We hypothesized that DPM-LPA would perform better on both metrics, producing more interpretable profiles than conventional LPA. In addition, we compared DPM-LPA to conventional LPA and finite Bayesian LPA in a simulation study to determine how accurately these different methods could infer the true number of latent profiles. We hypothesized that DPM-LPA would perform at least as well as the other methods.

The second goal was to validate the DPM-LPA neurocognitive latent profiles in relation to externalizing behaviors. Participants complete measures of mental health symptoms and behaviors (Barch et al., 2018), including the Child Behavior Checklist (CBCL) measure of externalizing behaviors (Achenbach, 2009) and the Positive and Negative Urgency Scales (Cyders et al., 2007; Whiteside, Lynam, Miller, & Reynolds, 2005), which measure affective-based impulsive behavior. We used Bayesian methods to investigate the relationship among the neurocognitive profiles discovered by DPM-LPA and externalizing behaviors. We expected that profiles characterized by worse neurocognitive performance would have higher average levels of externalizing but given that we present a novel application of DPM-LPA, we did not have more specific hypotheses about the relationships among neurocognitive profiles and externalizing behaviors.

## Method

### Participants

Participants were youth included in the ABCD Study Data Release 5.0 with a complete set of neurocognitive variables at the 2-year follow-up (T2; ages 11–12) (*n* = 5232; 47.9% Female, 52.1% Male; 1.9% Asian; 11.0% Black; 18.9% Hispanic; 10.2% Other; 57.9% White). We compared participants with a complete set of neurocognitive variables to those with missing neurocognitive data with respect to sex, race/ethnicity, externalizing behaviors, and internalizing behaviors (see Supplemental Material for details). The only difference was with respect to race/ethnicity: the proportion of Black and Hispanic youth was lower among participants with complete neurocognitive data (Black: 11.0% vs. 18.2%, Hispanic: 18.9% vs. 21.4%). All parents or caregivers provided written informed consent and children provided verbal assent for participation in the study (Clark et al., 2018). See Garavan and colleagues (2018) for the ABCD Study baseline exclusion criteria.

### Assessments

**Figure 2:**
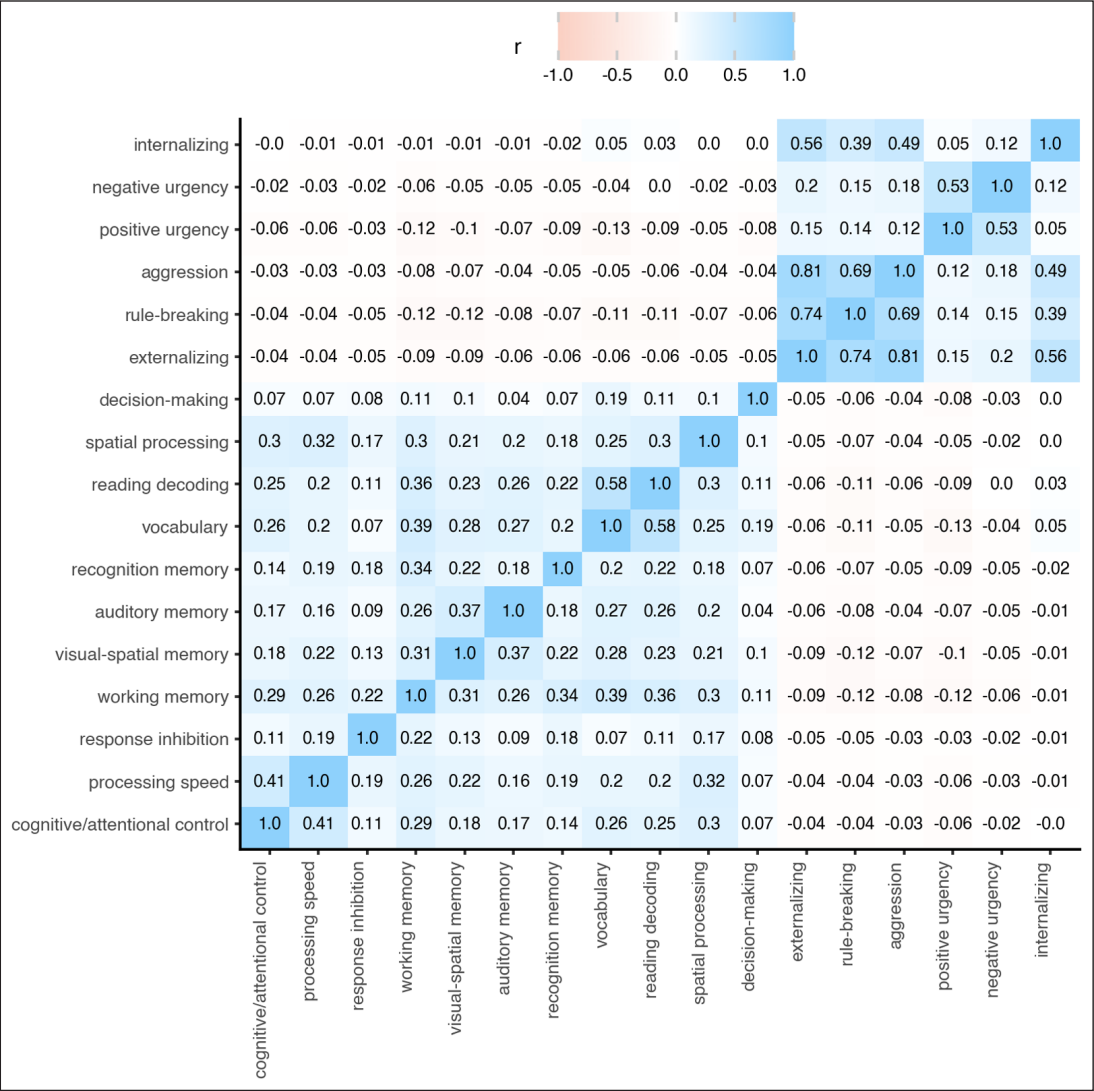
Correlations between variables (two year follow-up).

The ABCD Study data collection involves biannual visits an extensive evaluation across neurocognition and behavior (Jernigan, Brown, & Dowling, 2018). We used data from the 2-year and 3-year follow-up visits across all 21 ABCD sites (data release 5.0, doi: 10.15154/8873-zj65; https://nda.nih.gov/study.html?id=901). Figure 2 provides correlations among all variables.

#### Neurocognitive Data

We selected 11 neurocognitive measures (Table 1a) that spanned different domains of neurocognition, including executive functions, learning/memory, and general cognition (Thompson et al., 2019). For all NIH toolbox tasks, we used the uncorrected standardized scores to summarize behavioral performance given that there has been some question about the use of the T-scores for neurocognitive assessment in adolescents (Luciana et al., 2018; Taylor et al., 2022).

**Table 1:** Variables.


(A) NEUROCOGNITIVE (INDICATOR) VARIABLES (*x*)		

COGNITIVE PROCESS	TASK	DESCRIPTION

Executive Function		

cognitive/attentional control	Flanker (NIH Toolbox)	uncorrected standard score

processing speed	Pattern Comparison (NIH Toolbox)	uncorrected standard score

response inhibition	Stop Signal	–1× stop signal reaction time

working memory	Emotional N-Back	proportion correct on two-back trials

Learning and Memory		

visual-spatial memory	Picture Sequence Memory (NIH Toolbox)	uncorrected standard score

auditory memory	Rey Auditory Verbal Learning	immediate recall total correct

recognition memory	Emotional N-Back	recognition trials d′

General Cognition		

vocabulary	Picture Vocabulary (NIH Toolbox)	uncorrected standard score

reading decoding	Oral Reading Recognition (NIH Toolbox)	uncorrected standard score

spatial processing	Little Man Task	% correct/avg. correct response time

decision-making	Game of Dice	# safe choices – # risky choices

**(B) OUTCOME VARIABLES (y).**		

**VARIABLE**	**SAMPLE SIZE (2-YEAR FOLLOW-UP)**	**SAMPLE SIZE (3-YEAR FOLLOW-UP)**

externalizing (CBCL)	4977	4742

rule-breaking (CBCL)	4977	4742

aggression (CBCL)	4977	4742

internalizing (CBCL)	4977	4742

positive urgency (UPPS-P)	5230	–

negative urgency (UPPS-P)	5230	–


##### Executive functions

From the NIH Toolbox, we included Flanker (cognitive/attentional control) and Pattern Completion (processing speed). We also included a measure from the Stop Signal Task, which assesses response inhibition (Logan, 1994). On most trials, the participant’s objective is to quickly give one of two responses depending on the direction of an arrow. However, a subset of trials feature a stop signal, indicating that the response should be withheld. Analysis of response accuracy and response times allows computation of a stop signal reaction time (SSRT) that measures how well each participant can inhibit their responses; we used this measure (reverse coded by multiplying it by –1). Lastly, we included accuracy from the Emotional N-Back Test. The task includes 0-back blocks (respond to a stimulus seen at the beginning of the block) and 2-back blocks (respond to the stimulus seen two trials ago). The task includes both emotionally neutral stimuli (buildings) and emotionally charged stimuli (faces). We used accuracy across stimuli type during 2-back trials as a measure of working memory.

##### Learning and Memory

From the NIH Toolbox, we included Picture Sequence Memory (visuospatial sequencing/memory). To measure auditory memory, we used the number of correct words in the immediate recall test from the Rey Auditory Verbal Learning Task (Strauss, Sherman, & Spreen, 2006). Finally, in a second phase of the Emotional N-Back Test, a test of recognition memory was administered where participants are asked to discriminate between stimuli seen in the previous stage and new ones. We used *d*′, a discriminability metric from signal detection theory (Green & Swets, 1966) to measure recognition memory.

##### General Cognition

From the NIH Toolbox, we included Picture Vocabulary (measuring language skills/verbal intellect) and Oral Reading Recognition (language skills/reading decoding). We also used the Little Man Task (Acker & Acker, 1982) which measures visual-spatial processing. On each trial, participants see a rudimentary male figure rotated in various positions and must determine in which hand the figure is holding a briefcase. Performance is summarized by an efficiency score computed as percent correct responses divided by average response time. Lastly, to assess decision-making, we included the Game of Dice Task (Brand et al., 2005), which was adapted from the Iowa Gambling Task. On each trial, the participant chooses between a low-risk/low reward gamble and a high-risk/high-reward gamble. High-risk gambles are designed to be disadvantageous, so decision-making performance is measured by the number of low risk choices minus the number of high risk choices.

This set of neurocognitive measures differed from those examined in previous work using the ABCD data (Chaku et al., 2022; Thompson et al., 2019). We analyzed data from when participants were 11–12 years old, whereas similar previous work looked at baseline data (9–10 years old). Over that period, the set of neurocognitive tasks administered to ABCD participants changed: the Card Sort (cognitive flexibility) and List Sort (working memory) tasks were dropped after the baseline assessment, and the Game of Dice Task (decision-making) was added starting at the two-year-follow-up timepoint. In addition, we used behavioral data from the Stop Signal and Emotional N-Back tasks. All neurocognitive variables were standardized by subtracting the mean and dividing by the standard deviation (i.e. z-scored) prior to analysis.

#### Outcome Variables

We used the parent-report CBCL (Achenbach, 2009) and the UPPS (Cyders et al., 2007; Whiteside, Lynam, Miller, & Reynolds, 2005) to measure externalizing behaviors (Table 1b). The CBCL provides a total externalizing score, and two subscales representing rule- breaking and aggressive behaviors. The CBCL was collected at the two-year-follow-up timepoint (concurrent with the neurocognitive data) and at the three-year-follow-up timepoint (one year after the neurocognitive data). We also performed a sensitivity analysis including the broad internalizing measure from the CBCL (Achenbach, 2009), which measures anxiety, depression/withdrawal, and somatic complaints. We opted to use the parent-report for these measured because the youth-report assessment for the ABCD Study protocol changed across the timepoints (Barch et al., 2018), especially in the externalizing measures. In addition, research has documented good concordance between youth- and parent-report measures of externalizing disorders, which tend to be observable behaviors (note: concordance for internalizing tends to be lower than for externalizing in this age group; De Los Reyes & Kazdin, 2005). The UPPS scale provides two measures of impulsivity in affective circumstances (UPPS-Negative Urgency: the tendency to act rashly when experiencing negative emotions; UPPS-Positive Urgency: tendency to act rashly when experiencing positive emotion). The UPPS was only collected at the two-year-follow-up timepoint. All outcome variables were standardized by subtracting the mean and dividing by the standard deviation (i.e. z-scored).

### Latent Profile Analysis

#### Conventional Latent Profile Analysis (LPA)

We used the tidyLPA and mclust packages in R to perform conventional LPA (Rosenberg, Beymer, Anderson, Van Lissa, & Schmidt, 2019; Scrucca, Fraley, Murphy, & Raftery, 2023). We fit models with numbers of profiles ranging from 1 to 20. While it is rare to fit a conventional LPA model with 20 profiles, DPM-LPA can infer a large number of latent profiles if needed to describe the data, so including large conventional LPA models made for a fairer comparison. In mathematical terms, LPA is a finite mixture of multivariate Gaussians with a shared diagonal covariance matrix. The model starts by assuming that there is a fixed number of latent profiles (*T*) and that each individual belongs to a single latent profile. We use *z*_*i*_ to represent person *i*’s latent profile. For example, if person *i* belongs to latent profile 3, then *z*_*i*_ = 3. Latent profiles can be arbitrarily relabeled, so it is convention to label the profile containing the largest number of participants as profile 1, the next largest profile as profile 2, etc. Each person has a set of *m* observed indicator variables that we denote *x*_*i*_ = *x*_1,*i*_, *x*_2,*i*_, …, *x*_m,*i*_. In our study, these indicator variables are performance measures on the various neurocognitive tasks described in Table 1a. Each person’s indicator variables (*x*_*i*_) depend on the latent profile that person belongs to (*z*_*i*_). In particular, each indicator variable (*x*_*j,i*_) is assumed to have a normal distribution with a mean (*μ*_*j,t*_) that varies based on latent profile:


1 \[{x_{j,i}}{\mathrm{|}}{z_i} = t \sim {\cal N}({\mu _{j,t}},{\textstyle{1 \over {{\xi _j}}}})\qquad\quad i = 1,\,\,2,\,\, \ldots,\,\,n\quad j = 1,\,\,2,\,\, \ldots,\,{\mathrm{ }}m\]

Each profile is thus defined by its vector of means (*μ_t_* = *μ*_1,*t*_, *μ*_2,*t*_, …, *μ_m,t_*). Because all of our indicator variables are standardized (i.e. they have mean 0 and variance 1), it means that if *μ_j,t_* > 0 then people in profile *t* tend to have above average values of indicator variable *x*_*j*_,_*i*_, while if *μ_j,t_* < 0 they have below average values.

*ξ_j_* represents the precision, i.e. inverse variance, of indicator variable *j*: \[{\mathrm{var}}({x_{j,i}}) = {\textstyle{1 \over {{\xi _j}}}}\]. Thus, a variable with high precision has low variance, and vice versa (parameterization in terms of precision makes computations more convenient). We assume that precision (variance) does not differ across latent profiles. This is largely a pragmatic assumption: if precision did vary across profiles, it would be difficult to estimate it for smaller profiles (those with fewer people). We also assume that the indicator variables (*x*_*j,i*_) do not have any covariance with each other, i.e., they are independent given latent profile membership (*z*_*i*_). This is important for making latent profile analysis easy to interpret: one only needs to know a participant’s latent profile to know the distribution of any of their indicator variables (*x*_*j,i*_). The model has the following parameters that must be inferred:


2 \[{\xi_j} = {\mathrm{precision\ (inverse\ variance)\ of\ indicator}}\ j\qquad j = 1,\,\,2,\,\, \ldots,\,\,m\]

3 \[{\mu _{j,t}} = {\mathrm{mean\ of\ indicator}}\ j\ {\mathrm{in\ profile}}\ t\qquad j = 1,\,\,2,\,\, \ldots,\,\,m,\qquad \,t = 1,\,\,2,\,\, \ldots,\,\,T\]

4 \[{\pi _t} = {\mathrm{base\ rate\ probability\ of\ profile}}\ t\ \qquad t = 1,\,\,2,\,\, \ldots,\,\,T\]

This conventional LPA model is estimated using the expectation maximization (EM) algorithm (Dempster, Laird, & Rubin, 1977) to estimate these parameters by maximizing the model likelihood. This also produces estimates of *z* (profile membership) in the form of probability vectors (*ϕ*_*i*_):


5 \[{\phi _i} = [{\phi _{1,i}},\,\,{\phi _{2,i}},\,\, \ldots,\,\,{\phi _{T,i}}] = {\mathrm{probabilities\ that\ participant}}\ i\ {\mathrm{is\ in\ each\ profile}}\]

The conventional LPA model assumes a fixed number of latent profiles (*T*). Thus to determine how many latent profiles are the data one must fit multiple models with different values of *T* and determine which best describes the data. We examine three standard criteria for comparing models to choose the correct number of profiles. First, the entropy reduction statistic quantifies how confident each model is about its classification of people into different latent profiles. We describe it in more detail below. A low entropy reduction statistic (less than about 0.8) suggests that the model cannot accurately assign participants to latent profiles (Wang, Deng, Bi, Ye, & Yang, 2017). Second, the Akaike Information Criterion (AIC, Akaike, 1974) and Bayesian Information Criterion (BIC, Schwarz, 1978) measure goodness of fit (log likelihood) penalized by model complexity (number of estimated parameters). Lower values indicate better model fit balanced by model complexity. Finally, the Bootstrap Likelihood Ratio Test (Langeheine, Pannekoek, & Van de Pol, 1996) provides a test of whether – for each number of profiles – adding an additional profile improves model fit.

#### Dirichlet Process Mixture Latent Profile Analysis (DPM-LPA)

DPM-LPA is a non-parametric Bayesian form of LPA in which the number of latent profiles is not specified beforehand. Conventional LPA finds point estimates of model parameters by maximizing the likelihood of the data. In contrast, Bayesian models are fit by computing a probability distribution over their parameters (including participant-specific variables such as latent profile membership, *z*_*i*_) rather than a single point estimate. This distribution, the posterior distribution, combines a prior distribution (representing what parameter values are probable before data are observed) and the likelihood via Bayes’ Rule:


6 \[\overbrace {p({z_{1:n}},\,\,{\mu _{1:T}},\,\,{\xi _{1:m}}{\mathrm{|}}{x_{1:n}})}^{{\mathrm{posterior}}} = \frac{{\overbrace {p({x_{1:n}}{\mathrm{|}}{z_{1:n}},\,\,{\mu _{1:T}},\,\,{\xi _{1:m}})}^{{\mathrm{likelihood}}}\overbrace {p({z_{1:n}},\,\,{\mu _{1:T}},\,\,{\xi _{1:m}})}^{{\mathrm{prior}}}}}{{\underbrace {p({x_{1:n}})}_{{\mathrm{evidence}}}}}\]

Thus, in Bayesian statistics, parameter estimates are influenced by both the likelihood (as in conventional LPA) and the prior distribution. The prior distribution often leads to simpler model fits than would be obtained by maximum likelihood estimation (Jefferys & Berger, 1992).

DPM-LPA uses the same likelihood function as conventional LPA (Equation 1): each person’s vector of observed indicator variables (*x*_*i*_) follows a normal distribution with a mean vector (*μ*) that depends on the latent profile that person belongs to (*z*_*i*_) and with precisions (*ξ*) that are shared across profiles.

The prior distribution for DPM-LPA can be broken into two parts, the prior over latent profile membership, and the prior over means/precisions:


7 \[\overbrace {p({z_{1:n}},\,\,{\mu _{1:T}},\,\,{\xi _{1:m}})}^{{\mathrm{full\ prior}}} = \overbrace {p({z_{1:n}})}^{{\mathrm{profile\ membership}}}\overbrace {p({\mu _{1:T}},\,\,{\xi _{1:m}}{\mathrm{|}}{z_{1:n}})}^{{\mathrm{means/precisions}}}\]

**Figure 3:**
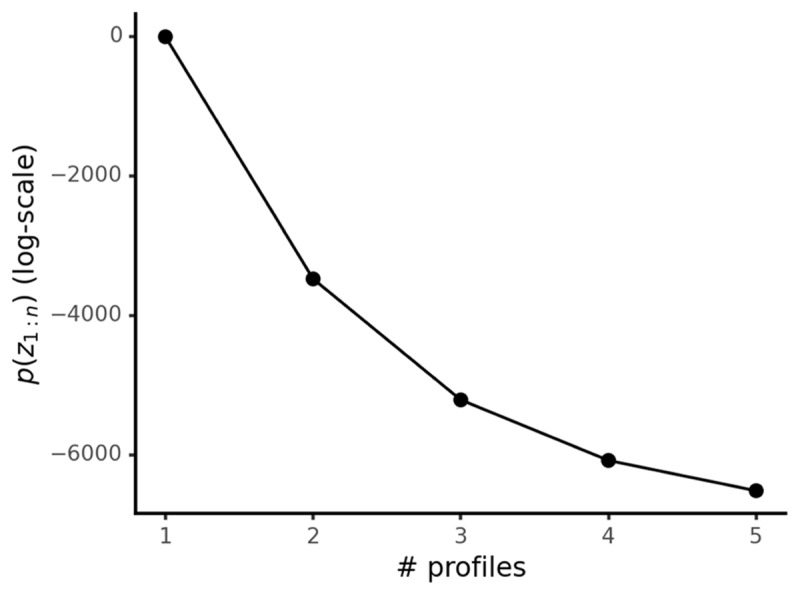
DPM-LPA prior distribution (log-scale) on participant latent profile membership (*z*_1:*n*_) as a function of the number of latent profiles. Calculated with *n* = 5000 participants, with each new profile created by splitting the last one in half.

The key to DPM-LPA is its prior on profile membership (*z_i_*), which is a distribution called the Dirichlet process (Ferguson, 1973). Using the Pólya Urn scheme representation (Blackwell & MacQueen, 1973), we can write it as:


8 \[p({z_{1:n}}) = \frac{{{\alpha ^T}\mathop \Pi \nolimits_{t = 1}^T ({n_t} - 1)!}}{{\alpha (\alpha + 1) \cdots (n - 1 + \alpha)}}{\mathrm{ }}\,\,\,\,\,{n_t} = {\mathrm{number\ of\ participants\ in\ profile}}\ t\]

where *α* is a concentration parameter (we infer *α* from the data, following Blei & Jordan, 2006). This Dirichlet process prior distribution does not have a hard upper limit on the number of latent profiles. However, it favors simpler LPA solutions. The prior on *z*_1:*n*_ is high when participants are concentrated in a few latent profiles and low when they are spread across several large ones, with this preference varying as a function of *α* (Figure 3). A more complex model fit, with participants assigned to a greater number of latent profiles, will only be favored (have a higher posterior probability) if the resulting increase in likelihood by adding those profiles outweighs the decrease in the prior distribution.

Means/precisions have a standard form of prior (called a conjugate prior) that keeps computations simple:


9 \[p({\mu _{1:T}},\,\,{\xi _{1:m}}{\mathrm{|}}{z_{1:n}}) = \mathop \prod \limits_{j = 1}^m \,\,p({\xi _j})\mathop \prod \limits_{t = 1}^T \,\,p({\mu _{j,t}}{\mathrm{|}}{\xi _j})\]

10 \[{\xi _j} \sim {\mathrm{Gamma}}(5,\,\,5){\mathrm{ }}\,\,\,\,j = 1,\,\,2,\,\, \ldots,\,\,m\]

11 \[{\mu _{j,t}}{\mathrm{|}}{\xi _j} \sim {\cal N}(0,{\textstyle{2 \over {{\xi _j}}}}){\mathrm{ }}\,\,\,\,j = 1,\,\,2,\,\, \ldots,\,\,m\,\,\,\,\,t = 1,\,\,2,\,\, \ldots,\,\,T\]

where *T* represents the number of distinct latent profiles implied by *z*_1:*n*_.

DPM-LPA is complex enough that its posterior distribution must be approximated. Markov chain Monte Carlo is one common approximation technique that produces a set of random samples from the posterior distribution (Ishwaran & James, 2001; Qiu et al., 2023). However, this is computationally expensive and thus not attractive for large datasets. Instead, we used an approximation method called mean field variational Bayes (for a more thorough discussion see Blei & Jordan, 2006; Blei, Kucukelbir, & McAuliffe, 2017). We implemented this algorithm and supporting code in Python. Once the DPM-LPA model has been fit, it is easy to determine how many latent profiles describe the data. Most of the latent profiles estimated by the model will be empty, i.e. the total estimated probability of any participants belonging to them will be approximately zero. We used the simple rule that if a profile was not estimated as maximally probable for at least one participant, that profile was considered to be empty and excluded from further analysis and discussion.

#### Entropy Reduction Statistic

We used a standard entropy reduction statistic to compare how confidently the conventional LPA and DPM-LPA models classify people into latent profiles. For each participant, the model estimates the probability of that person belonging to each latent profile: we call this *ϕ*_*i*_. For example, if for a certain participant *ϕ*_*i*_ = [0.9, 0.07, 0.03] then the model estimates that the probability of this person belonging to profile 1 is 90%, to profile 2 is 7%, and to profile 3 is 3% (in this example there are three latent profiles). Intuitively, the model is fairly confident that the participant is in profile 1. If the probabilities were closer to being equal, say for example *ϕ*_*i*_ = [0.4, 0.32, 0.28], then the model would be less confident about which profile the participant belonged to. The entropy of *ϕ*_*i*_ quantifies this uncertainty:


12 \[{\mathrm{entropy\ for\ participant}}\ i = - \mathop \sum \limits_{t = 1}^T \,\,{\phi _{t,i}}\,{\mathrm{log }}({\phi _{t,i}})\]

Consider the entropy of three hypothetical participants. In the first case (*ϕ*_1_ = [0.9, 0.07, 0.03]) entropy is low (entropy = 0.39), meaning that the model is fairly certain. In the second case (*ϕ*_2_ = [0.4, 0.32, 0.28]) entropy is high (entropy = 1.09), meaning that the model is not very certain. Maximum entropy occurs when all the values in *ϕ*_*i*_ are equal, meaning intuitively that the model does not have any idea which profile the participant belongs to (*ϕ*_3_ = [0.33, 0.33, 0.33], entropy = 1.10 in a model with three profiles).

The proportional entropy reduction statistic quantifies the overall certainty of a model. It compares the total entropy of a model across participants ( \[ - \Sigma _{i = {\mathrm{1}}}^n\;\Sigma _{t = 1\;}^T{\phi _{t,i}}\,{\mathrm{log }}({\phi _{t,i}})\]) to the maximum possible entropy ( \[ - n\,{\mathrm{log}}({\textstyle{1 \over T}})\]). Subtracting this ratio from 1 gives the following statistic:


13 \[{\mathrm{entropy\ reduction}} = \frac{{{\mathrm{max\ entropy}} - {\mathrm{total\ entropy}}}}{{{\mathrm{max\ entropy}}}} = 1 - \frac{{{\mathrm{total\ entropy}}}}{{{\mathrm{max\ entropy}}}} = 1 - \frac{{\mathop \sum \nolimits_{i = 1}^n \;\mathop \sum \nolimits_{t = 1}^T {\mathrm{ }}{\phi _{t,i}}{\mathrm{log (}}{\phi _{t,i}}{\mathrm{)}}}}{{n{\mathrm{log(}}{\textstyle{1 \over T}}{\mathrm{)}}}}\]

Values can range from 1 (total confidence in classifying participants) down to 0 (the opposite).

#### Profile Distinctiveness (Mahalanobis Distance)

Ideally, the latent profiles discovered by LPA should be very distinct from one another. This makes the profiles easier to interpret and reduces uncertainty about participants’ profile membership. We used the Mahalanobis distance to quantify the distinctiveness between each pair of profiles. This is a metric based on the Euclidean distance between two profiles’ mean vectors (*μ*_1_ and *μ*_2_), scaled by the precision, i.e. inverse variance (*ξ*):


14 \[{\mathrm{distance}} = \sqrt {\mathop \sum \limits_{j = 1}^m \,\,{\xi _j}{{({\mu _{j,1}} - {\mu _{j,2}})}^2}}\]

A distance of 0 indicates that two profiles are identical, while distance increases as profiles become more and more distinct. For each model (conventional LPA of varying sizes and DPM-LPA) we computed the minimum distance (to find the two most similar profiles in each model) as well as the mean distance across profile pairs.

### Simulations

We compared how well DPM-LPA, finite Bayesian LPA, and conventional LPA could infer the true number of latent profiles in simulated data. The simulation parameters were a subset of those used in a study by Tein, Coxe, and Cham (2013) examining various criteria for determining the number of profiles in conventional LPA. See the Supplemental Material for further details.

### Analyzing the Relationship between Latent Profiles and Outcome Variables

#### Estimating Profile Means ( \[{\mu ^{(y)}}\])

We used Bayesian methods to analyze the relationship between DPM-LPA latent profiles and outcome variables. For the sake of clarity, let us refer to the set of indicator variables (in our case neurocognitive measures) as *x* and the outcome as *y*. We assume that outcomes (e.g., CBCL externalizing) follow a normal distribution with a mean ( \[{\mu ^{(y)}}\]) that depends on the latent profile but a shared precision (*ξ*_*y*_):


15 \[{y_i}{\mathrm{|}}{z_i} = t \sim {\cal N}(\mu _t^{\left(y \right)},\,\,{\textstyle{1 \over {{\xi ^{\left(y \right)}}}}}){\mathrm{ }}i = 1,\,\,2,\,\, \ldots,\,\,n\]

We used a normal-gamma conjugate prior distribution:


16 \[{\xi ^{\left(y \right)}} \sim {\mathrm{Gamma(}}1,{\mathrm{ }}1{\mathrm{)}}\]

17 \[\mu _t^{\left(y \right)}{\mathrm{|}}{\xi ^{\left(y \right)}} \sim {\cal N}(0,{\textstyle{1 \over {{\xi ^{\left(y \right)}}}}}){\mathrm{ }}t = 1,\,\,2,\,\, \ldots,\,\,T\]

If each participant’s true latent profile (*z*_*i*_) was known, then the formula for estimating the outcome means ( \[{\mu ^{(y)}}\]) for each latent profiles would be standard conjugate prior updates. However, we of course do not know *z*_*i*_, but only have an estimate from the DPM-LPA fit in the form of a probability vector (*ϕ*_*i*_). We followed a simple, commonly used procedure (e.g. Chaku et al., 2022) and assigned participants to their most probable profiles:


18 \[{\mathrm{estimated\ profile\ for\ participant}}\ i = {\hat z_i} = \mathop {{\mathrm{argmax}}}\limits_{t = 1,\,2,\, \ldots,\,T} ({\phi _{t,i}}){\mathrm{ }}i = 1,\,\,2,\,\, \ldots,\,\,n\]

We then use these profile estimates ( \[{\hat z_i}\]) in the ordinary formulas to obtained approximate posterior distributions over each each outcome’s variance and profile means. This also gave us 95% posterior credible intervals over these parameters (the interval between the 2.5th and 97.5th percentiles of the posterior distribution).

#### Comparing Profile Means

We used a Bayesian analogue of the analysis of variance (ANOVA) to test if any of the profiles differed from each other, and if they did, we followed up with Bayesian post-hoc tests to determine which profiles had different means. The Bayesian ANOVA (Rouder, Morey, Speckman, & Province, 2012) compares a null hypothesis *H*_0_ (which assumes that all profiles have equal means) to the alternative hypothesis *H*_1_ that profile means differ:


19 \[{H_0}:\mu _1^{(y)} = \mu _2^{(y)} = \ldots = \mu _T^{(y)}\]

20 \[{H_1}:\mu _1^{(y)},\mu _2^{(y)}, \ldots,\mu _T^{(y)}{\mathrm{are\ not\ all\ equal}}\]

We compared these two hypotheses using the Bayes factor (*BF*_10_), which is defined as the ratio between the evidence for the two hypotheses:


21 \[B{F_{10}} = \frac{{p({y_{1:n}}{\mathrm{|}}{H_1})}}{{p({y_{1:n}}{\mathrm{|}}{H_0})}}\]

Where the evidence for either hypothesis is computed as the likelihood of the observed data (*y*_1_, *y*_2_, …, *y*_n_) averaged across the prior distribution:


22 \[p({y_{1:n}}{\mathrm{|}}{H_0}) = \mathop \smallint \nolimits_{{\xi ^{(y)}}} \mathop \smallint \nolimits_{{\mu ^{(y)}}} p({y_{1:n}}{\mathrm{|}}{\xi ^{(y)}},\,\,{\mu ^{(y)}})p({\xi ^{(y)}})p({\mu ^{(y)}})d{\mu ^{(y)}}d{\xi ^{(y)}}\]

23 \[p({y_{1:n}}{\mathrm{|}}{H_1}) = \mathop \smallint \nolimits_{{\xi ^{(y)}}} \mathop \smallint \nolimits_{\mu _1^{(y)}} \ldots \mathop \smallint \nolimits_{\mu _T^{(y)}} p({y_{1:n}}{\mathrm{|}}{\xi ^{(y)}},\,\,\mu _1^{(y)},\,\, \ldots,\,\,\mu _T^{(y)})\,\,p({\xi ^{(y)}})\,p(\mu _1^{(y)}) \ldots p(\mu _T^{(y)})d\mu _T^{(y)} \ldots d\mu _1^{(y)}d{\xi ^{(y)}}\]

Bayes factors greater than 1 show support for *H*_1_, Bayes factors less than 1 show support for *H*_0_, and Bayes factors close to 1 show similar amounts of evidence for both hypotheses.

Because Bayes factors can vary so widely in size, we also reported them in base-10 logarithmic scale:


24 \[{\mathrm{lo}}{{\mathrm{g}}_{10}}(B{F_{10}}) = {\mathrm{lo}}{{\mathrm{g}}_{10}}(p({y_{1:n}}{\mathrm{|}}{H_1})) - {\mathrm{lo}}{{\mathrm{g}}_{10}}(p({y_{1:n}}{\mathrm{|}}{H_0}))\]

On this logarithmic scale, positive values indicate support for *H*_1_, negative values indicate support for *H*_0_, and 0 indicates no support for either hypothesis. By convention (Kass & Raftery, 1995), if –0.5 < log_10_ (*BF*_10_) < 0.5 then the result is considered inconclusive: there is not substantial evidence for either hypothesis. We used the BayesFactor package (Morey & Rouder, 2022) in R for these calculations.

One way to accomplish post-hoc comparisons would be to use a Bayesian analogue of the t-test to test for equality between each pair of profile means. However, this approach suffers from two problems. First, each Bayesian t-test would only use the data from the two latent profiles being compared. This omits data from the other profiles, which is useful for estimating the outcome variance. Also, when there are more than three or four profiles, reporting and interpreting the results of all pairwise tests would be cumbersome.

Instead, we used a different post-hoc analysis method that arrives at a similar result to pairwise comparisons without suffering the same drawbacks (see Rouder et al., 2012). Each pairwise comparison can have two conclusions: the means are either equal or unequal. What results from the post-hoc analysis is a partitioning of the latent profiles into two or more sets, with equal profile means within each set and different means between sets. For a given number of latent profiles, we can enumerate all possible partitions (and thus all possible post-hoc analysis results) through a mathematical algorithm (Hutchinson, 1963). For example, with three latent profiles the post-hoc analysis could result in the following possible partitions:


\[\begin{array}{l}
\mu _1^{(y)} \ne \mu _2^{(y)};\,\,\mu _1^{(y)} \ne \mu _3^{(y)};\,\,\mu _2^{(y)} = \mu _3^{(y)} \to \{ 1\},\,\,\{ 2,\,\,3\} \\
\mu _1^{(y)} = \mu _2^{(y)};\,\,\mu _1^{(y)} \ne \mu _3^{(y)};\,\,\mu _2^{(y)} \ne \mu _3^{(y)} \to \{ 1,\,\,2\},\,\,\{ 3\} \\
\mu _1^{(y)} \ne \mu _2^{(y)};\,\,\mu _1^{(y)} = \mu _3^{(y)};\,\,\mu _2^{(y)} \ne \mu _3^{(y)} \to \{ 1,\,\,3\},\,\,\{ 2\} \\
\mu _1^{(y)} \ne \mu _2^{(y)};\,\,\mu _1^{(y)} \ne \mu _3^{(y)};\,\,\mu _2^{(y)} \ne \mu _3^{(y)} \to \{ 1\},\,\,\{ 2\},\,\,\{ 3\}
\end{array}\]

where the notation {1, 2}, {3} indicates that profiles 1 and 2 form a set with one shared mean, while profile 3 has a different mean and thus forms another set by itself.

Thus, instead of conducting pairwise Bayesian t-tests, we can enumerate all possible partitions of the latent profiles and decide which one describes the data best by comparing their model evidences (the calculation is the same as the Bayesian ANOVA).

#### Effect Sizes

We also computed a simple effect size measure for the relationship between the neurocognitive latent profiles and outcome variables. This was an *r*^2^ statistic similar to the one used with linear regression. First, we computed each individual’s predicted value ( \[{\hat z_i}\]) based on the fitted DPM-LPA model:


25 \[{\hat y_i} = \mu _{[{{\hat z}_i}]}^{(y)} = {\mathrm{mean\ of\ participant}}\ i^{\prime}{\mathrm{s\ profile}}\]

Then we computed *r*^2^ as the proportional reduction in error:


26 \[{r^2} = \frac{{\mathop \sum \nolimits_{i = 1}^n {{({y_i} - \bar y)}^2} - \mathop \sum \nolimits_{i = 1}^n {{({y_i} - {{\hat y}_i})}^2}}}{{\mathop \sum \nolimits_{i = 1}^n {{({y_i} - \bar y)}^2}}} = 1 - \frac{{\mathop \sum \nolimits_{i = 1}^n {{({y_i} - {{\hat y}_i})}^2}}}{{\mathop \sum \nolimits_{i = 1}^n {{({y_i} - \bar y)}^2}}}\]

where \[\bar y\] is the overall mean of *y*.

## Results

### Simulations

DPM-LPA has similar performance to conventional LPA and superior performance to finite Bayesian LPA (see Supplemental Figure 1). DPM-LPA detected the correct number of latent profiles more often than conventional LPA (using either the BIC or BLRT) at a small sample size of *n* = 250 (DPM-LPA: 92% correct, conventional LPA with BIC: 82% correct, conventional LPA with BLRT: 72% correct). DPM-LPA was equally accurate to conventional LPA using the BLRT at larger sample sizes (*n* = 500, 1000), although the BIC was slightly more accurate (BIC: 98% correct, DPM-LPA/BLRT: 92–94% correct). Finite Bayesian LPA was less accurate than either DPM-LPA or conventional LPA, with the difference increasing at larger sample sizes.

### Selecting the Number of Profiles in Conventional LPA

**Figure 4:**
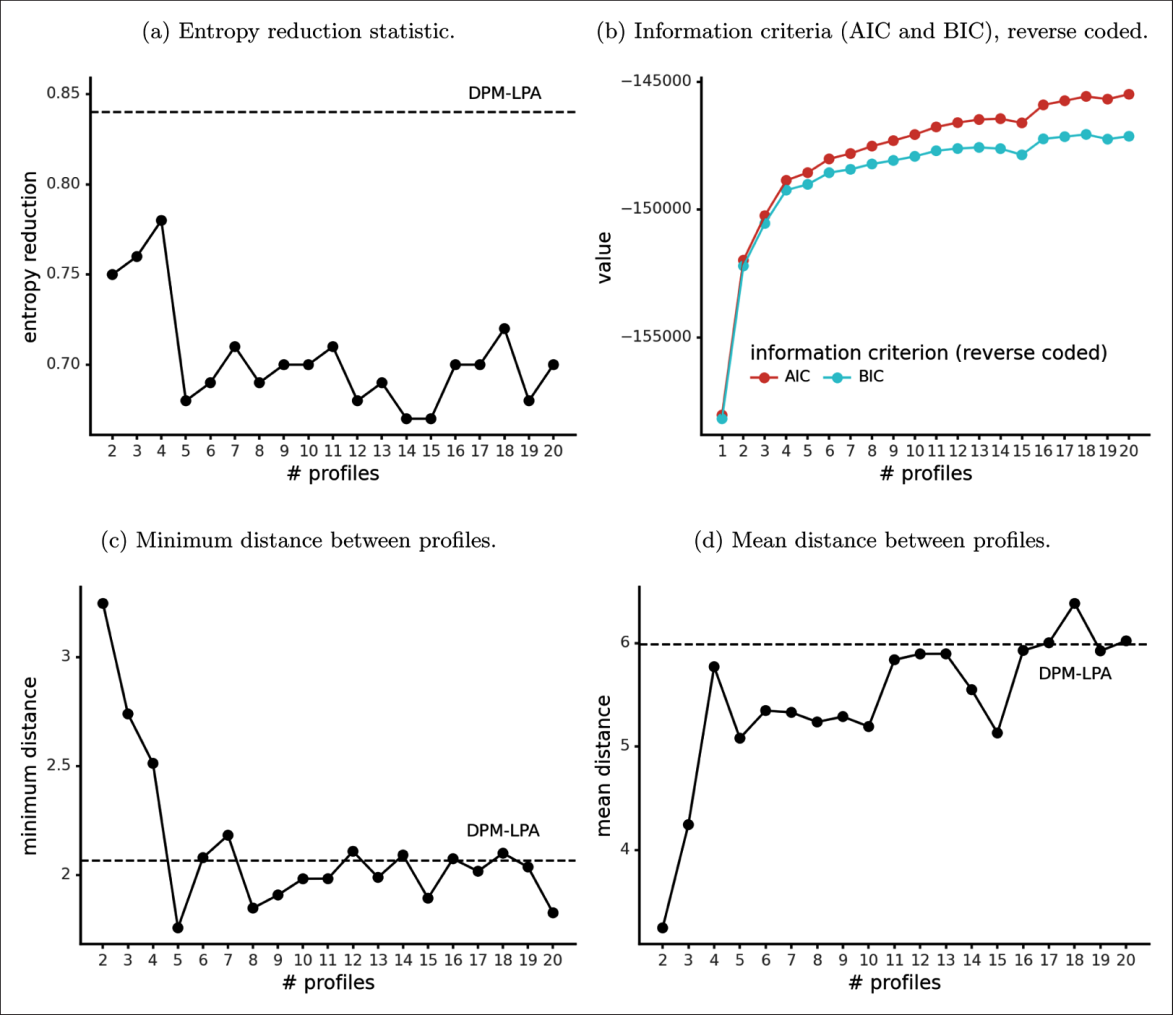
Comparisons of conventional LPA models with differing numbers of profiles (all variables are coded so that larger means better). Where applicable, a dashed horizontal line indicates the corresponding value from DPM-LPA.

Commonly used criteria (entropy reduction statistic, AIC, BIC, bootstrap likelihood ratio test) led to contradictory conclusions about the correct number of latent profiles in our sample. The entropy reduction statistic became worse (i.e., decreased) as the number of profiles increased (Figure 4a), falling to low levels (around 0.7) for models with more than 4 profiles. This suggested that the correct number of profiles is small. However, AIC, BIC, and the bootstrap likelihood test led to the opposite conclusion. AIC and BIC improved as the number of profiles increased (Figure 4b, note that AIC and BIC are reverse coded, i.e. multiplied by –1, so larger values are better), showing that adding more profiles improved model fit. Similarly, the bootstrap likelihood ratio test reported a statistically significant improvement in model fit (*p* < 0.01) with each profile added up to 16 profiles.

### Comparing Conventional LPA and DPM-LPA: Entropy Reduction Statistic

DPM-LPA had a better entropy reduction statistic than any conventional LPA model (Figure 4a). In other words, DPM-LPA was more confident than conventional LPA in its classification of participants into latent profiles. This may be due to the relative distinctiveness of DPM-LPA’s profiles, as described above.

### Comparing Conventional LPA and DPM-LPA: Profile Distinctiveness (Mahalanobis Distance)

In conventional LPA, smaller models (2, 3, or 4 profiles) had a higher minimum pairwise distance between profiles than the larger ones (Figure 4c). In contrast, mean pairwise distance between profiles was lower for the very small conventional LPA models (2 or 3 profiles) than the larger ones (Figure 4d). In general, DPM-LPA was comparable or better than conventional LPA models with 5 or more profiles on both measures of distinctiveness (minimum and mean pairwise distance). Small conventional LPA models (2, 3, or 4 profiles) had better minimum distance than DPM-LPA, but worse mean distance. DPM-LPA (which discovered 9 latent profiles) was superior to the 9-profile conventional LPA model on both measures.

### Description of Conventional LPA Latent Profiles

We selected the 4-profile model because its entropy reduction statistic was acceptable (0.78); larger models had unacceptably low entropy reduction statistics (0.67–0.72), despite their superior BIC/AIC and support from the BLRT. Based on these criteria, a four profile solution was selected (see Supplemental Figure 2): Profile 1 (56% of the sample) represented average neurocognition, Profile 2 (21% of the sample) represented above average neurocognition, Profile 3 (21% of the sample) represented below average neurocognition, and Profile 4 (2% of the sample) represented below average neurocognition with exceptionally low working memory.

### Description of DPM-LPA Latent Profiles

**Figure 5:**
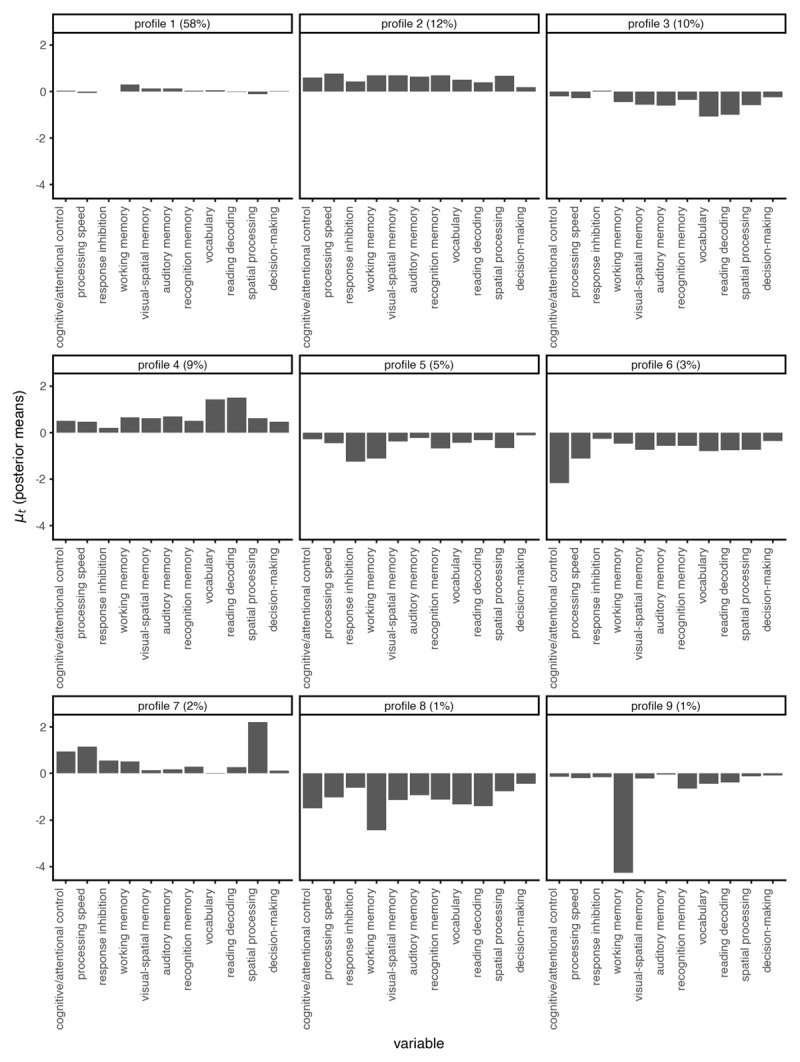
Estimated latent profile means (*μ*) from DPM-LPA.

DPM-LPA identified nine latent profiles. Figure 5 shows plots of the estimated means for each profile. Profile 1 represented average performance across neurocognitive components and encompassed 58% of the sample. Profile 2 (12% of the sample) represented above average performance across most neurocognitive components. Profile 3 (10% of the sample) represented below average performance across most neurocognitive components, with particularly low vocabulary and reading decoding but average response inhibition. Participants in Profile 4 (9% of the sample) had broadly above average neurocognitive function, with particularly good vocabulary and reading decoding. Participants in Profile 5 (5% of the sample) had below average working memory, recognition memory, and response inhibition. Profile 6 (3% of the sample) was characterized by low cognitive/attentional control. Profile 7 (2% of the sample) was characterized by above average processing speed, cognitive/attentional control, and spatial processing. Profiles 8 and 9 each contained 1% of the sample. Profile 8 showed generally poor neurocognitive functioning, while participants in Profile 9 had average performance on most tasks but exceptionally low working memory. Overall, profiles 2, 4, and 7 represented different patterns of above average neurocognitive function, while profiles 3, 5, 6, 8, and 9 represented different patterns of below average neurocognitive function. Importantly, each profile represented a unique distribution of neurocognitive scores.

### Neurocognitive Latent Profiles and Psychopathology

**Figure 6:**
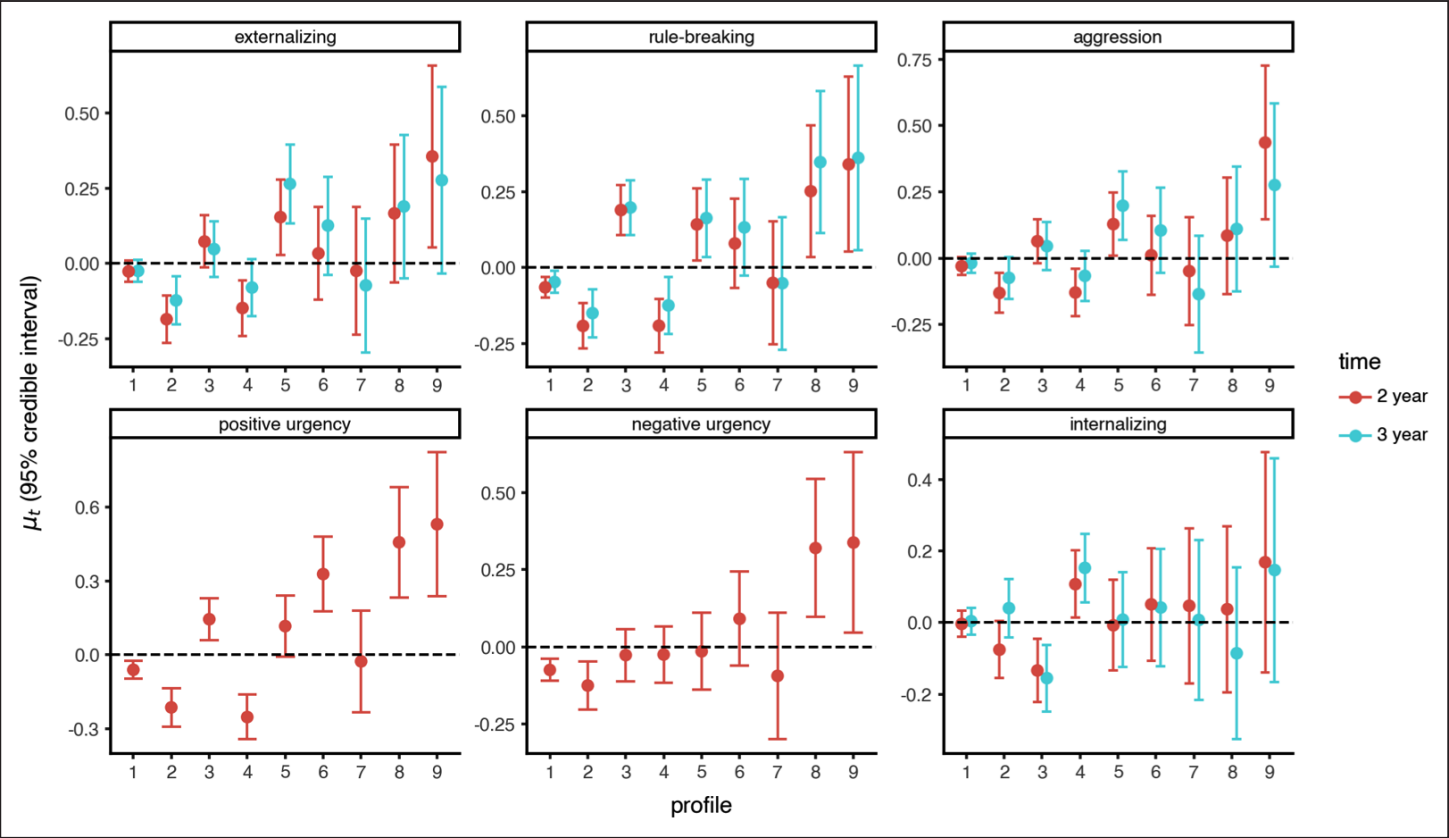
Estimated DPM-LPA latent profile outcome means (95% posterior credible interval).

See Figure 6 for plots of estimated profile means for all outcome variables and Table 2 for a summary of results. For comparison with DPM-LPA, we conducted a similar analysis using the 4-profile conventional LPA model (Supplemental Table 2). Results using conventional LPA were broadly similar to the DPM-LPA results reported below, but the DPM-LPA analysis provided a more nuanced description of the relationship between neurocognition and externalizing behaviors.

**Table 2:** Summary of outcome analysis results. log_10_ (*BF*10) is the Bayes factor for the Bayesian ANOVA on a base-10 logarithmic scale. *r*^2^ is the effect size. \[\mu _j^{(y)}\] is the mean value of the relevant outcome variable for profile *j*.


(A) TWO YEAR FOLLOW-UP.			

VARIABLE	log10 (BF_10_)	*r* ^2^	POST-HOC RESULTS

externalizing	3.51	0.0090	\[\mu _2^{(y)} = \mu _4^{(y)} < \mu _1^{(y)} = \mu _6^{(y)} = \mu _7^{(y)} < \mu _3^{(y)} = \mu _5^{(y)} = \mu _8^{(y)} = \mu _9^{(y)}\]

rule-breaking	11.59	0.0167	\[\mu _2^{(y)} = \mu _4^{(y)} < \mu _1^{(y)} = \mu _7^{(y)} < \mu _3^{(y)} = \mu _5^{(y)} = \mu _6^{(y)} = \mu _8^{(y)} = \mu _9^{(y)}\]

aggression	1.22	0.0070	\[\mu _2^{(y)} = \mu _4^{(y)} < \mu _1^{(y)} = \mu _6^{(y)} = \mu _7^{(y)} < \mu _3^{(y)} = \mu _5^{(y)} = \mu _8^{(y)} < \mu _9^{(y)}\]

positive urgency	18.87	0.0230	\[\mu _2^{(y)} = \mu _4^{(y)} < \mu _1^{(y)} = \mu _7^{(y)} < \mu _3^{(y)} = \mu _5^{(y)} < \mu _6^{(y)} = \mu _8^{(y)} = \mu _9^{(y)}\]

negative urgency	–0.47	0.0052	NA (inconclusive)

internalizing	–1.57	NA	NA (no differences)

**(B) THREE YEAR FOLLOW-UP.**			

**VARIABLE**	**log10 (BF_10_)**	***r* ^2^**	**POST-HOC RESULTS**

externalizing	2.11	0.0079	\[\mu _2^{(y)} = \mu _4^{(y)} = \mu _7^{(y)} < \mu _1^{(y)} = \mu _3^{(y)} < \mu _5^{(y)} = \mu _6^{(y)} = \mu _8^{(y)} = \mu _9^{(y)}\]

rule-breaking	8.01	0.0140	\[\mu _2^{(y)} = \mu _4^{(y)} < \mu _1^{(y)} = \mu _7^{(y)} < \mu _3^{(y)} = \mu _5^{(y)} = \mu _6^{(y)} = \mu _8^{(y)} = \mu _9^{(y)}\]

aggression	–0.85	NA	NA (no differences)

internalizing	–0.62	NA	NA (no differences)


#### Externalizing

##### Externalizing, Rule-Breaking, and Aggression

Overall externalizing behaviors at the two-year follow-up differed across latent profiles ( \[{\mathrm{lo}}{{\mathrm{g}}_{10}}(B{F_{10}}) = 3.51,\,\,B{F_{10}} = 3.22\,\, \times \,\,{10^3},\,\,{r^2} = 0.0090\]). Post-hoc tests showed that profiles 6 and 7 had equal means to profile 1 (average neurocognition), profiles 2 and 4 were related to lower levels of externalizing, while profiles 3, 5, 8, and 9 were related to higher levels of externalizing.

The rule-breaking and aggression subscales also differed across latent profiles (rule-breaking: log_10_ (*BF*_10_) = 11.59, *BF*_10_ = 3.87 × 10^11^, *r*^2^ = 0.0167; aggression: log_10_ (*BF*_10_) = 1.22, *BF*_10_ = 16.70, *r*^2^ = 0.0070). However, the relationship with neurocognition was much stronger for rule-breaking (*r*^2^ = 0.0167) than aggression (*r*^2^ = 0.0070). Post-hoc tests for rule-breaking revealed that profile 7 (posterior mean estimate: \[\hat \mu _7^{(y)} = - 0.05\]) had the same mean as profile 1 (average neurocognition, \[\hat \mu _1^{(y)} = - 0.06\]), profiles 2 ( \[\hat \mu _2^{(y)} = - 0.19\]) and 4 ( \[\hat \mu _4^{(y)} = - 0.19\]) had a lower mean level of rule-breaking, and profiles 3 ( \[\hat \mu _3^{(y)} = 0.19\]), 5 ( \[\hat \mu _5^{(y)} = 0.14\]), 6 ( \[\hat \mu _6^{(y)} = 0.08\]), 8 ( \[\hat \mu _8^{(y)} = 0.25\]), and 9 ( \[\hat \mu _9^{(y)} = 0.34\]) showed higher mean levels of rule-breaking. For aggression, profiles 6 ( \[\hat \mu _6^{(y)} = 0.01\]) and 7 ( \[\hat \mu _7^{(y)} = - 0.05\]) had the same mean as profile 1 ( \[\hat \mu _1^{(y)} = - 0.03\]), profiles 2 ( \[\hat \mu _2^{(y)} = 0.13\]) and 4 ( \[\hat \mu _4^{(y)} = - 0.13\]) showed lower mean levels of aggression, profiles 3 ( \[\hat \mu _3^{(y)} = 0.06\]), 5 ( \[\hat \mu _5^{(y)} = 0.13\]), and 8 ( \[\hat \mu _8^{(y)} = 0.08\]) showed higher mean aggression, and profile 9 ( \[\hat \mu _9^{(y)} = 0.44\]) had the highest mean level of aggression.

At the three-year follow-up, the latent profiles were still related to overall externalizing behaviors (log_10_ (*BF*_10_) = 2.11, *BF*_10_ = 1.30 × 10^2^, *r*^2^ = 0.0079) and rule-breaking (log_10_ (*BF*_10_) = 8.01, *BF*_10_ = 1.03 × 10^8^, r^2^ = 0.0140), but not aggression (log_10_ (*BF*_10_) = –0.85, *BF*_10_ = 0.14). Post-hoc results for rule-breaking were the same as those for the two-year follow-up described above. However, the post-hoc results for overall externalizing changed at the three-year follow-up. Profiles 1 ( \[\hat \mu _1^{(y)} = - 0.02\]) and 3 ( \[\hat \mu _3^{(y)} = 0.05\]) shared the same mean externalizing, profiles 2 ( \[\hat \mu _2^{(y)} = - 0.12\]), 4 ( \[\hat \mu _4^{(y)} = - 0.08\]), and 7 ( \[\hat \mu _7^{(y)} = - 0.07\]) had a lower mean externalizing, and profiles 5 ( \[\hat \mu _5^{(y)} = 0.26\]), 6 ( \[\hat \mu _6^{(y)} = 0.13\]), 8 ( \[\hat \mu _8^{(y)} = 0.19\]), and 9 ( \[\hat \mu _9^{(y)} = 0.28\]) had a higher mean externalizing.

##### Positive and Negative Urgency

Positive urgency differed across neurocognitive latent profiles (log_10_ (*BF*_10_) = 18.87, *BF*_10_ = 7.46 × 10^18^, *r*^2^ = 0.0230). Post-hoc tests showed that profile 7 ( \[\hat \mu _7^{(y)} = - 0.03\]) had the same positive urgency mean as profile 1 ( \[\hat \mu _1^{(y)} = - 0.06\]), profile 2 ( \[\hat \mu _2^{(y)} = - 0.21\]) and profile 4 ( \[\hat \mu _4^{(y)} = - 0.25\]) had a lower positive urgency mean, profile 3 ( \[\hat \mu _3^{(y)} = 0.14\]) and profile 5 ( \[\hat \mu _5^{(y)} = 0.12\]) had a higher positive urgency mean, while profiles 6 ( \[\hat \mu _6^{(y)} = 0.33\]), 8 ( \[\hat \mu _8^{(y)} = 0.46\]), and 9 ( \[\hat \mu _9^{(y)} = 0.53\]) had the highest positive urgency mean. The Bayes factor testing the relationship between neurocognitive profiles and negative urgency was indecisive (log_10_ (*BF*_10_) = –0.47, *BF*_10_ = 0.34, *r*^2^ = 0.0052); if there is a relationship, it is weaker than for positive urgency (negative urgency: *r*^2^ = 0.0052 vs. positive urgency: *r*^2^ = 0.0230).

##### Interim Summary

Altogether, profiles 2 and 4 seem to capture a group of adolescents typified by above average neurocognitive performance and good behavioral regulation capacities across the two-and-three-year data (consistently had the lowest levels of overall externalizing behaviors, rule-breaking, and aggression). In contrast, profiles 3, 5, 6, 8 and 9 showed below average performance across most neurocognitive measures, and overall had higher than average levels of externalizing behaviors. Profile 3 included adolescents who struggled the most with tasks that rely on vocabulary and reading skills and display higher than average levels of externalizing, rule-breaking, and aggression (although their level of externalizing declined to the same level as profile 1, the average group, at the three-year follow-up). Profile 5 captured adolescents who showed relatively poorer executive functions (working memory and response inhibition), which related to their externalizing behaviors. Adolescents in profile 6 showed a particular pattern of neurocognitive scores that indicates that they found it challenging to engage cognitive/attentional control, which was related to higher scores on rule-breaking and positive urgency but not aggression. Finally, profiles 8 and 9 appear to capture the most severe cases of general neurocognitive problems, particularly in terms of working memory, and these groups showed higher levels externalizing behaviors across measures and time.

#### Internalizing

Sensitivity analysis showed that the neurocognitive profiles were not related to the CBCL internalizing scale at either the two-year-follow-up (log_10_(*BF*_10_) = –1.57, *BF*_10_ = 0.03) or the three-year-follow-up (log_10_(*BF*_10_) = –0.62, *BF*_10_ = 0.24). This suggest that the differences in neurocognitive patterns within-persons are specific to externalizing-related outcomes (see also Chaku et al., 2022).

## Discussion

The overarching goal of this work was to develop and test a novel approach to study the relationship between individual differences in neurocognitive functioning and externalizing behaviors in adolescents. Our findings highlight the advantages and power of a novel non-parametric Bayesian approach to LPA, called DPM-LPA, for untangling subgroups of adolescents based on an extensive and diverse set of neurocognitive metrics, even when groups overlap in the patterns of observable externalizing behaviors.

### Comparing LPA Methods

Consistent with previous work (e.g. Chaku et al., 2022; Wendt et al., 2019), different selection criteria gave different answers about the correct number of profiles in conventional LPA (Figure 4). Model fit (measured by AIC and the bootstrap likelihood ratio test) improved by increasing the number of profiles, but this came at the cost of having highly similar, redundant profiles and hence lower classification certainty (measured by the entropy reduction statistic). This illustrates the tradeoff in conventional LPA between model fit and model interpretability.

In contrast, DPM-LPA automatically decided how many latent profiles to include. Compared to conventional LPA models with equal flexibility, DPM-LPA produced less similar profiles and classified participants into profiles with greater certainty (i.e., had better entropy reduction, Figure 4a). This can be explained by DPM-LPA’s non-parametric Bayesian inference process. Fitting conventional LPA only involves maximizing the likelihood, which rewards the fitting algorithm for using all available latent profiles to classify people even if some of them end up being very similar (Figure 4d). However, DPM-LPA takes both the likelihood and prior distribution into account. The prior favors a small number of profiles that actually contain participants, leaving unneeded profiles empty. Thus, DPM-LPA has the flexibility to infer a large number of latent profiles if it needs to, but does not tend to infer redundant, highly similar profiles, in the way that conventional LPA does. Simulations provide further support for DPM-LPA (see Supplemental Material): it compares well with conventional LPA in terms of detecting the correct number of latent profiles, and may be the superior method at small sample sizes (*n* = 250).

### Neurocognitive Latent Profiles and Externalizing Behaviors

In addition to methodological superiority over conventional LPA, DPM-LPA also provides a general approach to generate unique insights into how the same externalizing behaviors can be associated with differences in patterns of neurocognition across subgroups. Broadly speaking, in the present study, the profiles showing more externalizing behaviors were typified by below average neurocognitive functioning, and those characterized by above average neurocognitive performance showed the opposite pattern. However, most importantly, we were able to further pinpoint subgroup-specific patterns reflecting issues with subcomponents of executive functions (working memory, inhibition) and language processing in the latent profiles showing relatively higher levels of externalizing.

The presence of subgroups predominantly showing reduced working memory capacity is in line with the proposal that executive function plays a key role in the development of reduced behavioral regulation during childhood (Moffitt, 1993; Zelazo, 2020). Notably, we also identified subgroups with equally worse performance on two subcomponents of executive functions, working memory and inhibition (profile 5), and two out of three subgroups showing poorer working memory also presented with worse inhibition (profiles 8 and 9). This pattern suggests a possible interaction between these two subcomponents of executive functions, which converges with prior evidence in adults indicating that cognitive inhibition accounts for part of the variability in age-related working memory (Borella, Carretti, & De Beni, 2008). In sum, these findings further highlight the sensitivity of the DPM-LPA for detecting latent profiles based on fine-grained differences in neurocognitive functions, perhaps even providing sufficient sensitivity to disentangle subgroups in which the interaction of different subcomponents of neurocognitive functions could turn out to be a key etiological factor. Future studies could explore this possibility further.

Our findings also highlight the existence of a group of adolescents with externalizing proneess and below average general neurocognitive functioning that especially struggle with language development (profile 3). Prior work has shown that language skills play a particularly important role in the development of externalizing behaviors, with one study indicating that having poorer language abilities is a temporally stable within-person predictor of having more externalizing problems later in childhood (Petersen & LeBeau, 2021). The importance of identifying which adolescents present with poor language abilities becomes particularly salient in the context of intervening on externalizing behaviors, as many interventions are delivered verbally and thus place a burden on individuals’ language skills. It may be necessary to support these individuals through alternative strategies that take into consideration their neurocognitive difficulties.

Importantly, our results suggest that the association between neurocognition and externalizing behaviors varies depending on the subgroup of neurocognition and subtype of externalizing behaviors. For example, we saw divergence in the association between variability in neurocognition and engagement in impulsive behavior when feeling positive versus negative emotions. Below average neurocognitive profiles all related to higher positive urgency (albeit to different degrees). There was little evidence of differences in neurocognition related to negative urgency. It is possible that for this age-group, emotion-relevant impulsivity, particularly positive emotions, relates to neurocognitive vulnerability. It also is possible that the association between neurocognitive difficulties and negative urgency emerges at more extreme levels of impulsive behavior (Stautz & Cooper, 2013). Far more work is needed to examine if neurocognitive associations differentially relate to positive and negative urgency (Johnson, Elliott, & Carver, 2020; Johnson, Tharp, Peckham, Sanchez, & Carver, 2016; Littlefield, Stevens, Ellingson, King, & Jackson, 2016). As another example, we saw a lack of temporal stability in the association between neurocognitive profiles and aggressive behavior measured at timepoints two (age 11–12) and three (age 12–13). This finding is consistent with developmental research which points out that aggression and rule-breaking represent different dimensions within the context of broad externalizing behaviors, each with a separate pattern of (neuro)biological correlates and developmental timeline (Burt, 2013; Harden et al., 2015). While aggression decreases as children age, the tendency to break rules increases over time and into adolescence (Bongers, Koot, Van Der Ende, & Verhulst, 2004). Follow-up studies in future waves of the ABCD data collection could try to elucidate the developmental changes in neurocognition and behavioral outcomes.

### Limitations

This study had several limitations which can be addressed in future work. First, based on the ABCD two-year follow-up neurocognitive data we find that DPM-LPA is superior to conventional LPA. Examining the robustness of this claim with other datasets, varying in sample type, measures of neurocognition, and the number of variables will be an important next step.

Second, while the neurocognitive battery used at the two-year-follow-up is quite extensive, it did not tap all neurocognitive functions (e.g., cognitive flexibility) or include tasks with more ecologically-valid stimuli (Conley & Baskin-Sommers, 2023; Young, Frankenhuis, DelPriore, & Ellis, 2022). Therefore, the pattern of neurocognitive functions may shift when considering additional cognitive and task-related factors. Related, adolescence is a time of rapid neurocognitive and behavioral development. Therefore, we may obtain very different neurocognitive latent profiles at an older or younger age. Future work should investigate the stability or change in neurocognitive latent profiles and their relationship to externalizing behaviors.

Third, our DPM-LPA software is limited in several ways and can be improved upon in later versions. The software can currently only model indicator variables using normal distributions. However, many psychological applications of LPA involve binary or categorical indicators, or indeed a combination of variable types. Implementing greater flexibility in the allowable distributions of indicators would improve the applicability of DPM-LPA (e.g. Qiu et al., 2023).

Finally, our simulations comparing DPM-LPA to conventional LPA and finite Bayesian LPA were limited by the empirical focus of the current study. Simulations for evaluating statistical methods require substantial computational resources, and are often full studies in and of themselves (e.g. Tein et al., 2013). We only simulated one possible scenario (10 indicator variables, 5 latent profiles, equal number of participants in each latent profile, profile means separated by 1.5 standard deviations) out of many possible ones. We also only generated 50 simulated data sets in each condition: more replications could improve our estimates of each model/method’s accuracy. Further, detecting the correct number of latent profiles is not the only criterion by which to compare methods: accuracy of assigning participants to the correct latent profiles and accuracy of estimating profile means also are important. Conducting extensive simulations that address these limitations would be a useful direction for future research.

## Conclusions

This paper presents a novel implementation of DPM-LPA, a non-parametric Bayesian approach to conducting LPA, which can detect latent profiles from relatively large amounts of indicator variables and offers a possible solution for dealing with the tradeoff between interpretability and model fit that plagues conventional LPA. We showed that DPM-LPA can be used to better understand how externalizing behaviors seen across individuals relate to differences in neurocognitive subcomponents by detecting latent subgroups of individuals with similar neurocognition. Our study marks a step towards addressing the challenge of finding novel ways to use data on neurocognitive functioning to better describe the individual (Brazil et al., 2018; Ruiz et al., 2023). Such advances are particularly relevant given that current call for person-centered diagnostics and tailored interventions for individuals showing these costly behaviors.

## Additional File

The additional file for this article can be found as follows:

10.5334/cpsy.112.s1 Supplemental material. Comparison of participants with complete and incomplete data, simulations, conventional LPA outcome analysis.
