# Supplementary material for: Enhancing Within-Person Estimation of Neurocognition and the Prediction of Externalizing Behaviors in Adolescents
Source: Comput Psychiatr. 2024 Jul 26;8(1):119–41. doi: 10.5334/cpsy.112 (PMC11276473; doi:10.5334/cpsy.112)
Supplement: Supplemental material. — Comparison of participants with complete and incomplete data, simulations, conventional LPA outcome analysis. [file cpsy-8-1-112-s1.pdf]

# Comparing Participants with Complete and Missing Neurocognitive Data

For sex and race/ethnicity we computed Bayes factors for independence in contingency tables (with total $N$ not fixed); for externalizing and internalizing behaviors we performed a Bayesian t-test (Bayes factors were computed using the BayesFactor package in R using default settings, Morey & Rouder, 2022). Sex was independent of neurocognitive data missingness ($BF_{10} = 0.00, \log_{10}(BF_{10}) = -3.53$), but race/ethnicity was not ($BF_{10} = 7.67 \times 10^{32}, \log_{10}(BF_{10}) = 32.88$). In particular, the proportion of Black and Hispanic youth was lower among participants with complete neurocognitive data (Black: 11.0% vs. 18.2%, Hispanic: 18.9% vs. 21.4%). Neither externalizing behaviors ($BF_{10} = 0.08, \log_{10}(BF_{10}) = -1.09$) nor internalizing behaviors ($BF_{10} = 0.02, \log_{10}(BF_{10}) = -1.60$) differed as a function of neurocognitive data missingness.

Table 1: Comparison of participants with complete and missing neurocognitive data.

(a) Sex.

| | % Female | % Male | % Intersex-Male |
|---|---|---|---|
| complete | 47.9 | 52.1 | 0.0 |
| missing | 47.8 | 52.1 | 0.1 |

(b) Race/ethnicity.

| | % Asian | % Black | % Hispanic | % Other | % White |
|---|---|---|---|---|---|
| complete | 1.9 | 11.0 | 18.9 | 10.2 | 57.9 |
| missing | 2.3 | 18.2 | 21.4 | 10.7 | 47.4 |

# Simulations

We simulated data with 5 latent profiles and 10 indicator variables, with an equal number of simulated participants from each latent profile. Within each latent profile, each indicator variable ($x$) had the same mean, i.e.

$$\mu_{1,t} = \mu_{2,t} = \ldots = \mu_{m,t} \quad \text{for each profile } t$$

Profile means were separated from each other at successive intervals of 1.5 standard deviations, i.e. $\mu_1 = -3.0$, $\mu_2 = -1.5$, $\mu_3 = 0.0$, $\mu_4 = 1.5$, and $\mu_5 = 3.0$, with error variance ($\sigma^2$) fixed at 1. We generated 50 data sets at each of three sample sizes: $n = 250$, 500, and 1000. Each of the three LPA models (DPM-LPA, finite Bayesian LPA, and conventional LPA) was fit to the same simulated data. DPM-LPA and conventional LPA were implemented as described above. We used the BIC and BLRT as criteria for inferring the correct number of latent profiles in conventional LPA based on their good performance in previous simulations (Tein, Coxe, & Cham, 2013). We implemented finite Bayesian LPA in Python using variational Bayes (the algorithm is very similar to the DPM-LPA algorithm), fitting a single over-fitted model (with 20 latent profiles) using a Dirichlet distribution prior hyperparameter less than 1 (we used 0.5) to eliminate redundant profiles (as suggested by White & Murphy, 2014); this is essentially the same procedure for determining the number of latent profiles that we used for DPM-LPA. Initial testing showed following a similar procedure to conventional LPA, viz. fitting finite Bayesian LPA models of varying sizes (1 profile, 2 profiles, 3 profiles etc.) and comparing them using the evidence lower bound (ELBO) to determine the correct number of latent profiles was so ineffective that it was not worth pursuing further.

Figure 1: Ability to determine the true number of latent profiles from simulated data.

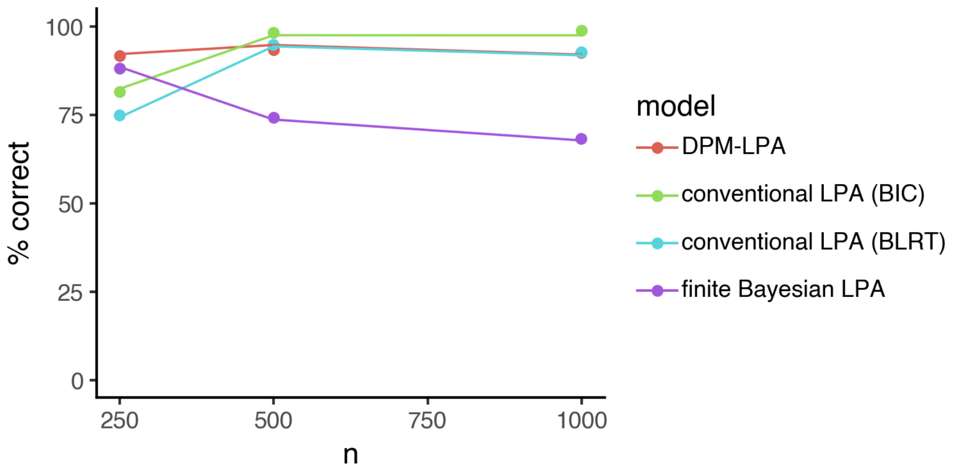


# Description of Conventional LPA Profiles

See Figure 2 for the profile means from conventional LPA (the 4-profile model). The profiles can be characterized as 1: average neurocognition, 2: above average neurocognition, 3: below average neurocognition, and 4: below average neurocognition with exceptionally low working memory.

Figure 2: Estimated latent profile means ($\mu$) from conventional LPA (with 4 profiles).

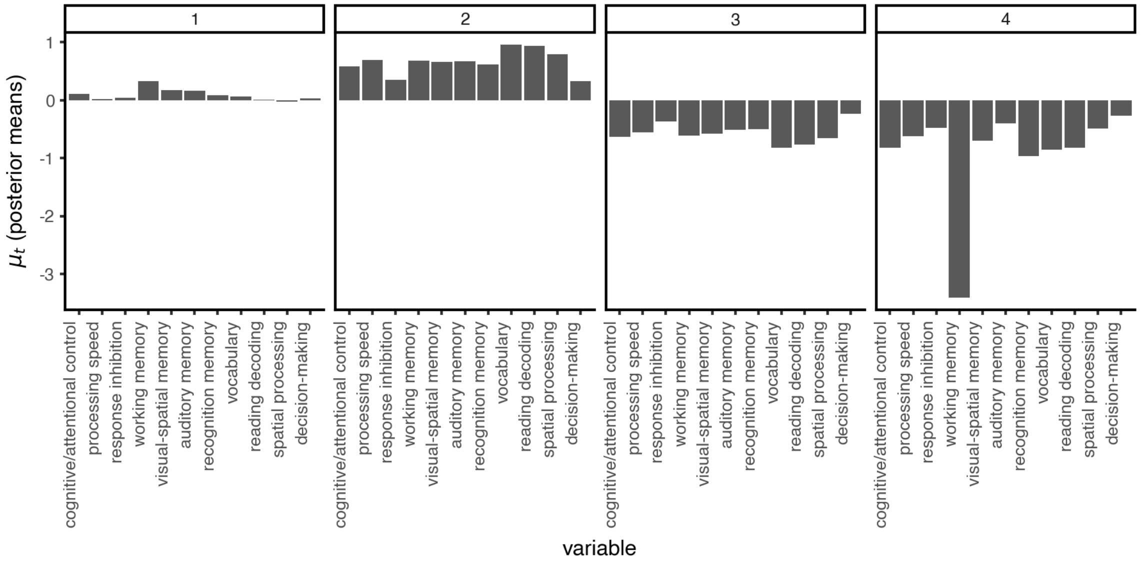

# Conventional LPA Outcome Analysis

See Table 2 for outcome analysis results. These results are largely consistent with DPM-LPA: poorer neurocognition is associated with higher externalizing behavior, but not internalizing behavior. However, the DPM-LPA analysis provided a more nuanced description of the relationship between neurocognition and externalizing behaviors.

Table 2: Summary of outcome analysis results for conventional LPA (4 profiles). $\log_{10}(BF_{10})$ is the Bayes factor for the Bayesian ANOVA on a base-10 logarithmic scale. $r^2$ is the effect size. $\mu_j^{(y)}$ is the mean value of the relevant outcome variable for profile $j$.

(a) Two year follow-up.

| Variable | $\log_{10}(BF_{10})$ | $r^2$ | Post-Hoc Results |
|---|---|---|---|
| externalizing | 5.26 | 0.0096 | $\mu_2^{(y)} < \mu_1^{(y)} < \mu_3^{(y)} = \mu_4^{(y)}$ |
| rule-breaking | 15.70 | 0.0198 | $\mu_2^{(y)} < \mu_1^{(y)} < \mu_3^{(y)} = \mu_4^{(y)}$ |
| aggression | 3.49 | 0.0074 | $\mu_2^{(y)} < \mu_1^{(y)} < \mu_3^{(y)} < \mu_4^{(y)}$ |
| positive urgency | 19.25 | 0.0222 | $\mu_2^{(y)} < \mu_1^{(y)} < \mu_3^{(y)} < \mu_4^{(y)}$ |
| negative urgency | 0.92 | 0.0041 | $\mu_1^{(y)} = \mu_2^{(y)} < \mu_3^{(y)} < \mu_4^{(y)}$ |
| internalizing | | NA | NA (no differences) |

(b) Three year follow-up.

| Variable | $\log_{10}(BF_{10})$ | $r^2$ | Post-Hoc Results |
|---|---|---|---|
| externalizing | 2.92 | 0.0075 | $\mu_1^{(y)} = \mu_2^{(y)} < \mu_3^{(y)} = \mu_4^{(y)}$ |
| rule-breaking | 11.13 | 0.0173 | $\mu_1^{(y)} = \mu_2^{(y)} < \mu_3^{(y)} = \mu_4^{(y)}$ |
| aggression | 0.61 | 0.0049 | $\mu_1^{(y)} = \mu_2^{(y)} < \mu_3^{(y)} = \mu_4^{(y)}$ |
| internalizing | -0.43 | 0.0022 | NA (inconclusive) |
